# Supplementary material for: Identification and Characterization of MiRNAs in Coccomyxa subellipsoidea C-169
Source: Int J Mol Sci. 2019 Jul 13;20(14):3448. doi: 10.3390/ijms20143448 (PMC6678167; doi:10.3390/ijms20143448)
Supplement: Supplementary file 1 [file ijms-20-03448-s001.zip › supplemental material/Supplemental Figure.docx]

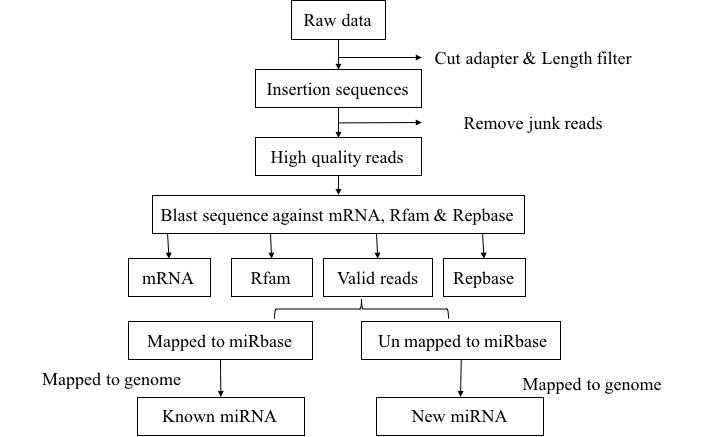


**Figure S1**. Pipeline used to identify C-169 miRNA.


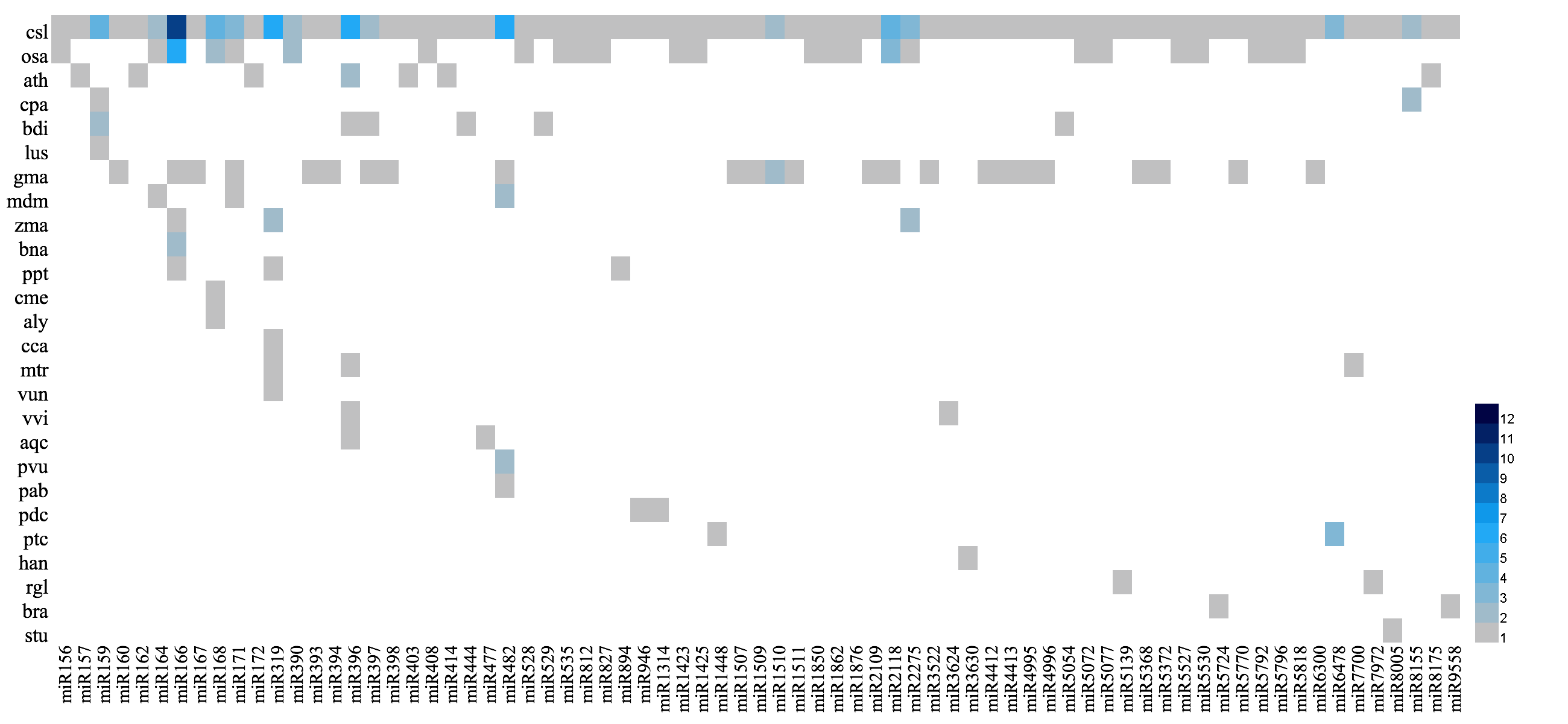


**Figure S2.** Distribution of identified conserved miRNA families of C-169 in different plant species. The color-coding is used to indicate the number of miRNA members in each family. aly-*Arabidopsis lyrata*, ath-*Arabidopsis thaliana*, aqc-*Aquilegia caerulea*, bdi-*Brachypodium distachyon*, bna-*Brassica napus*, bra-*Brassica rapa*, cca-*Cynara cardunculus*, cme-*Cucumis melo*, cpa-*Carica papaya*, gma-*Glycine max*, han-*Helianthus annuus*, lus-*Linum usitatissimum*, mdm-*Malus domestica*, mtr-*Medicago truncatula*, osa-*Oryza sativa*, pab-*Picea abies*, pde-*Pinus densata*, ppt-*Physcomitrella patens*, ptc-*Populus trichocarpa*, pvu-*Phaseolus vulgaris*, rgl-*Rehmannia glutinosa*, stu-*Solanum tuberosum*, vun-*Vigna unguiculata*, vvi-*Vitis vinifera*, zma-*Zea mays*).


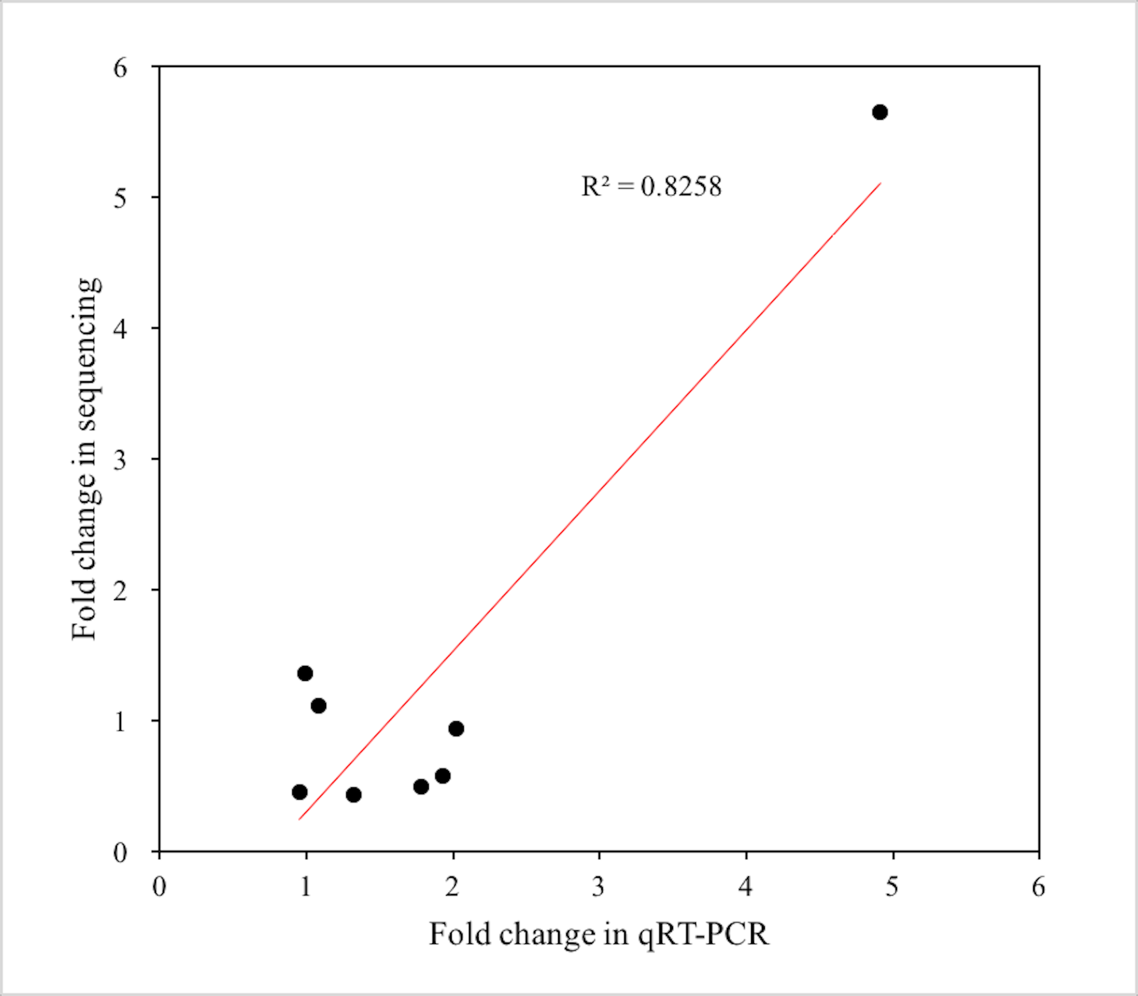


**Figure S3.** Sequencing data of differential expressed miRNAs were validated by quantitative RT-PCR





**Figure S4.** The expression ratio of miRNAs and their putative target genes upon CO_2_ supplementation (CG/AG) related to lipid metabolism. Orange bars represent miRNAs and the blue bars represent their putative target genes.
